# Simple questions on simple associations: regularity extraction in non-human primates

**DOI:** 10.3758/s13420-023-00579-z

**Published:** 2023-06-07

**Authors:** Jeremy Yeaton, Laure Tosatto, Joël Fagot, Jonathan Grainger, Arnaud Rey

**Affiliations:** 1 Aix Marseille Univ, CNRS, LPC, Marseille, France; 2 Department of Language Science, University of California – Irvine, 2243 Social Sciences Plaza, Irvine, CA 92617 USA; 3 Aix Marseille Univ, ILCB, Aix-en-Provence, France; 4 Station de Primatologie, CNRS-Celphedia, UPS 846, Rousset-sur-Arc, Rousset, France

**Keywords:** Statistical learning, Sequence learning, Associative learning, Animal cognition

## Abstract

When human and non-human animals learn sequences, they manage to implicitly extract statistical regularities through associative learning mechanisms. In two experiments conducted with a non-human primate species (Guinea baboons, *Papio papio*), we addressed simple questions on the learning of simple AB associations appearing in longer noisy sequences. Using a serial reaction time task, we manipulated the position of AB within the sequence, such that it could be either fixed (by appearing always at the beginning, middle, or end of a four-element sequence; Experiment 1) or variable (Experiment 2). We also tested the effect of sequence length in Experiment 2 by comparing the performance on AB when it was presented at a variable position within a sequence of four or five elements. The slope of RTs from A to B was taken for each condition as a measurement of learning rate. While all conditions differed significantly from a no-regularity baseline, we found strong evidence that the learning rate did not differ between the conditions. These results indicate that regularity extraction is not impacted by the position of the regularity within a sequence and by the length of the sequence. These data provide novel general empirical constraints for modeling associative mechanisms in sequence learning.

**Supplementary Information:**

The online version contains supplementary material available at 10.3758/s13420-023-00579-z.

## Introduction

A key building block of our cognitive lives is the ability to detect regularities between two events A and B that cooccur frequently in the environment. By repeatedly processing and encoding these AB regularities, we progressively manage to predict or expect B when A is appearing. These fundamental statistical learning abilities help us learn and execute complex sequences of information more rapidly and fluidly (Christiansen, 2019; Frost et al., 2019; Perruchet & Pacton, 2006).

Several computational models have been proposed to describe the mechanisms supporting these statistical learning abilities (e.g., Elman, 1990; Endress & Johnson, 2021; Frank, Goldwater, Griffiths, & Tenenbaum, 2010; French et al., 2011; Giroux & Rey, 2009; Perruchet & Vinter, 1998; Pothos, 2007; Tovar, Westermann, & Torres, 2018, Tovar & Westermann, 2023). Most of these models were developed to account for human data collected on artificial language learning tasks such as the one initially introduced in the seminal study by Saffran, Aslin, and Newport (1996). In this task, participants were exposed to languages generally composed of four to six trisyllabic artificial words that were concatenated without any pause between words. After being exposed to this language for several minutes, participants were able to discriminate words from other trisyllabic nonword sequences indicating that they extracted the underlying statistical information.

This crucial ability has also been studied in non-human primates like tamarins (e.g., Hauser et al., 2001), macaques (e.g., Wilson et al., 2015), chimpanzees (e.g., Sonnweber et al., 2015; Watson et al., 2020) and baboons (e.g., Malassis et al., 2018; Minier et al., 2016; Rey et al., 2019, 2022; Tosatto et al., 2022), suggesting that regularity extraction is supported by common associative learning mechanisms across these species (Rey et al., 2019). The advantage of studying these mechanisms in non-human primates is the absence of any interference related to language recoding processes that may blur the study of associative learning mechanisms.

Previous studies with Guinea baboons (*Papio papio*) have revealed several general properties of these associative mechanisms. These studies used a visuo-motor pointing task derived from the serial reaction time task (Nissen & Bullemer, 1987) in which baboons were expected to touch a moving target on a touch screen that could appear on nine possible positions on an evenly spaced three-by-three grid. It was found that when baboons were repeatedly exposed to regularities composed of three successive positions ABC, RTs on the third position of the regular triplet (i.e., C) were found to decrease faster than RTs on the second position (i.e., B) (Minier et al., 2016). Using sequences of three positions, it has also been reported that baboons were able to learn second-order regularities when first-order regularities were inconsistent (Rey et al., 2022). With longer sequences composed of nine positions, it has been shown that baboons are segmenting these long sequences into chunks of 3–4 positions, revealing fundamental limits of associative learning mechanisms (Tosatto et al., 2022).

Most of the studies conducted in human and non-human primates investigated their ability to extract statistical information about regularities composed of at least three adjacent ABC elements (i.e., trisyllabic words in artificial language learning tasks or triplets in visuo-motor pointing tasks). Paradoxically, less is known about much-simpler associations, like the repeated cooccurrence of AB regularities. Notably, there are three simple questions regarding these simple regularities for which we do not have empirical responses that may inform models of statistical learning.

The first question is related to the position of the AB regularity within a longer sequence. If AB always occurs at a specific ordinal position in a sequence, does it make a difference to extract that regularity when it occurs at the beginning, in the middle, or at the end of the sequence? Studies on short-term memory have identified several types of serial position effects indicating that the position of information in a sequence matters (e.g., Oberauer et al., 2018). Similarly, in the study of reading processes, crowding effects indicate that information situated inside a sequence receives more interference compared to information situated at the beginning or the end (e.g., Grainger, 2022). The extraction of an AB regularity may therefore depend on the ordinal position of the regularity with regularities situated inside the sequence being potentially harder to extract than the ones situated at the beginning or the end.

The second question concerns a different case in which the AB regularity is not always occurring at the same position but may appear at all possible positions in a sequence. Here the question is whether the extraction of these regularities is easier or more difficult than regularities appearing always at the same position in the sequence. The variable position of the regularity may slow down its extraction or the fixed position may instead facilitate its extraction because of the potential encoding of the ordinal position of the regularity that may provide an additional processing cue.

The third question concerns the length of the sequence in which the regularity is occurring. If the length of the sequence increases then the amount of information also increases and this modifies the signal-to-noise ratio since the signal (i.e., the regularity) is not embedded in the same amount of “noise”. We may therefore expect harder extraction of an AB regularity when it appears in longer sequences.

Therefore, our goal in the present study was to expand further our knowledge about the general properties of associative mechanisms in sequence learning and to address these three rather simple questions on the extraction of simple AB regularities. To answer these questions, we have conducted two experiments with Guinea baboons (*Papio papio*) and with the serial pointing task that has been extensively used to study their ability to extract regularities (e.g., Minier et al., 2016; Rey et al., 2022; Tosatto et al., 2022). As mentioned before, the advantage of testing non-human primates is the absence of possible recoding strategies that are related to the language faculty and that are of course absent in these species, leading to more direct investigations of the underlying associative learning mechanisms.

In the first experiment, baboons were exposed to sequences composed of a fixed length of four elements. An AB regularity systematically appeared at the same position within the sequence on each trial. The sequence of four positions was therefore composed of the AB regularity and two additional random elements (X) that were drawn from the seven remaining possible positions. Three conditions were tested: AB was either presented first, followed by the two random elements (ABXX condition), after two random elements (XXAB condition) or between the two random elements (XABX condition). Baboons were repeatedly administered one of the three conditions at a time each for 500 trials in order to compare the learning rates of AB in each condition. If the position of the regularity in the sequence had an effect on its learning, we expect differences in the decrease in RTs for the predicted B position as a function of its position in the sequence.

In a second experiment, we tested if the learning of an AB regularity would vary as a function of the sequence’s length. Baboons were either exposed to four-element sequences composed of the AB regularity and two random elements or to five-element sequences composed of the AB regularity and three random elements. Here, contrary to Experiment 1, AB was not associated to a specific position within the longer sequence and could appear at any position on each trial. We can therefore contrast the learning rates obtained in Experiment 1 in which AB appeared at a fixed position in sequences of four elements with the learning of AB when it appeared randomly at any position in the sequences of four (in Experiment 2). We can also contrast the learning rate of AB when it appeared in sequences of four or five elements.

## Experiment 1

### Method

#### Participants

We tested 20 Guinea baboons (*Papio papio*, 16 females, age range 2.92–25 years) living in a social group at the CNRS primate facility in Rousset, France. The baboons were members of a social group of 25 individuals living in a 700-m^2^ outdoor enclosure containing climbing structures connected to two indoor experimental areas containing the test equipment. Water was provided ad libitum during the test, and the monkeys received their normal food ration of fruits every day at 5:00 PM.

#### Apparatus

The baboons had free access to 14 Automated Learning Devices for Monkeys (ALDM, Fagot & Bonté, 2010; Fagot & Paleressompoulle, 2009) equipped with tactile screens and a food dispenser. Whenever a monkey entered a test chamber, it was identified by its microchip, and the system was prompted to resume the trial list at the place at which the subject left it at its previous visit. The experiment was controlled by E-Prime 2.0 (Psychology Software Tools, Pittsburgh, PA, USA) (Psychology Software Tools, Inc., 2016).

#### Materials and procedure

To initiate a trial, the baboon had to touch a yellow cross presented at the bottom of the screen. After the baboon touched it, the yellow cross disappeared, and nine white crosses were displayed, with one of them being replaced by the target, a red circle. When the target circle was touched, it disappeared and was immediately replaced by the cross. The next position in the sequence was then replaced by a second red target circle until the end of the sequence was reached. When the baboon successfully completed the sequence of touches, it was automatically delivered a reward (grains of dry wheat). If the baboon touched an incorrect location or failed to complete the trial within 5000 ms, a green screen was displayed for 3000 ms to indicate the trial had been failed.

**Fig. 1:**
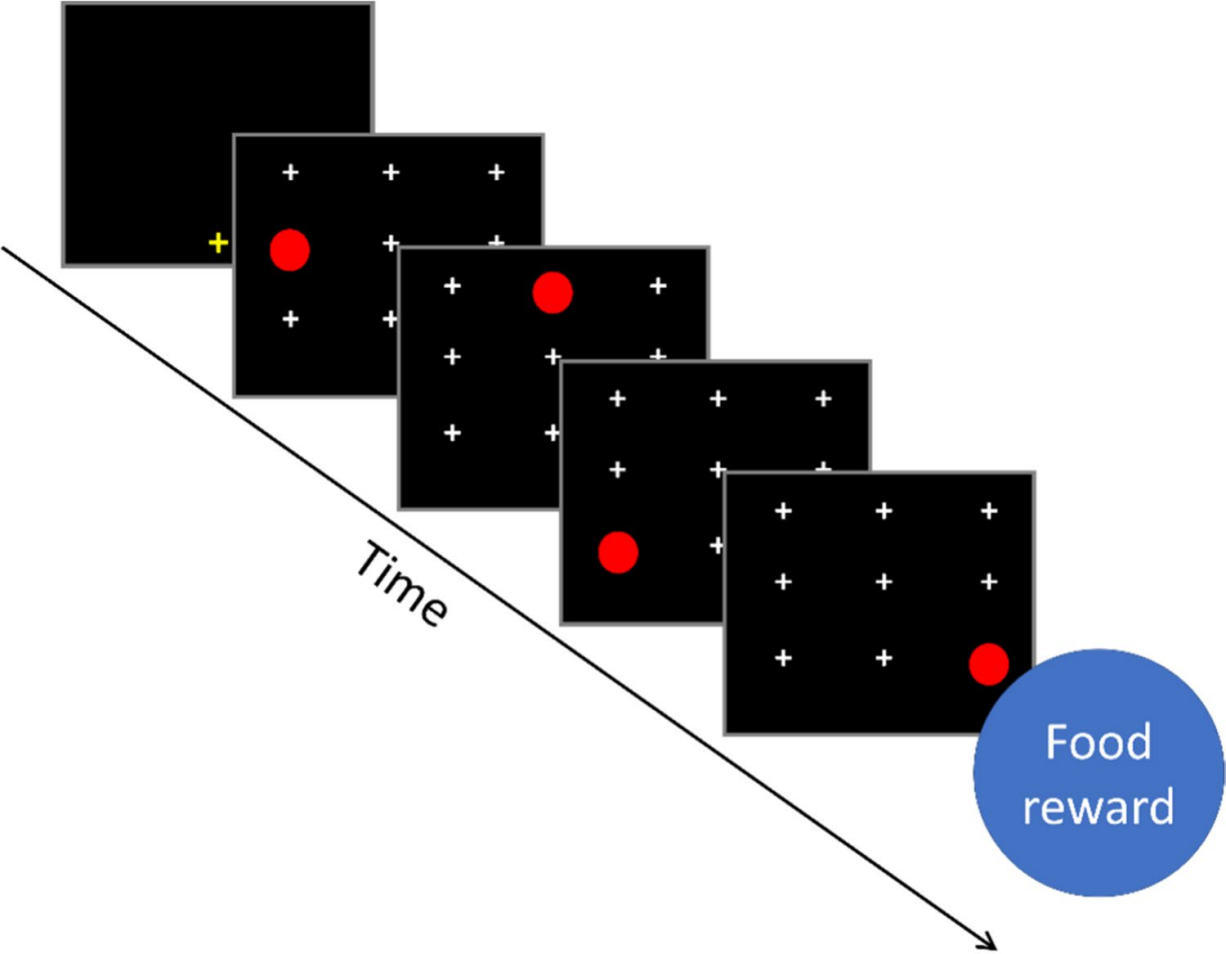
Schematic of a single trial in Experiment 1

The task began with a familiarization phase during which baboons were presented with random sequences of four positions. For each touch, the response time (RT) between the appearance of the circle and the baboon’s touch was recorded (Fig. 1). After 500 random trials, the baboon passed to the first block of experimental trials. They each saw three blocks of 500 trials each, one experimental condition being assigned to each block.

In all three conditions, each trial was composed of four touches: two forming the AB regularity which appeared on every trial (in the same position in the sequence), and two that were drawn uniformly from the seven positions not used in the regularity. For example, if the regularity was 5-1, these two positions would appear in the same order, adjacent to one another, on every trial, and 5 or 1 would not appear again in the sequence.

The position of the regularity in the sequence varied across the three experimental conditions: it appeared in the first position (ABXX), the second position (XABX), or the third position (XXAB). The order of the conditions was counterbalanced across baboons. To avoid learning effect across conditions, each baboon had a different regularity for each condition. These regularities were matched for difficulty using the baseline RTs collected during a previous task where the baboons were presented with 1000 random sequences composed of six touches. RTs for that task were averaged across all trials for each transition from one position to the next in the sequence. A baseline measure for all possible transitions from one position to another was obtained, yielding a 9 × 9 matrix of mean transition times (calculated over the entire group of monkeys, see Appendix A).

Three AB regularities were then assigned to each baboon with the following constraints. For each baboon, the three pairs could not have baseline RTs with a difference of greater than 10 ms. No position could be used twice in the three pairs for a given baboon (i.e., if a baboon was assigned the pair 5-1, neither 5 nor 1 could appear in any other pair). The three pairs used for each baboon are presented in Appendix B.

**Fig. 2:**
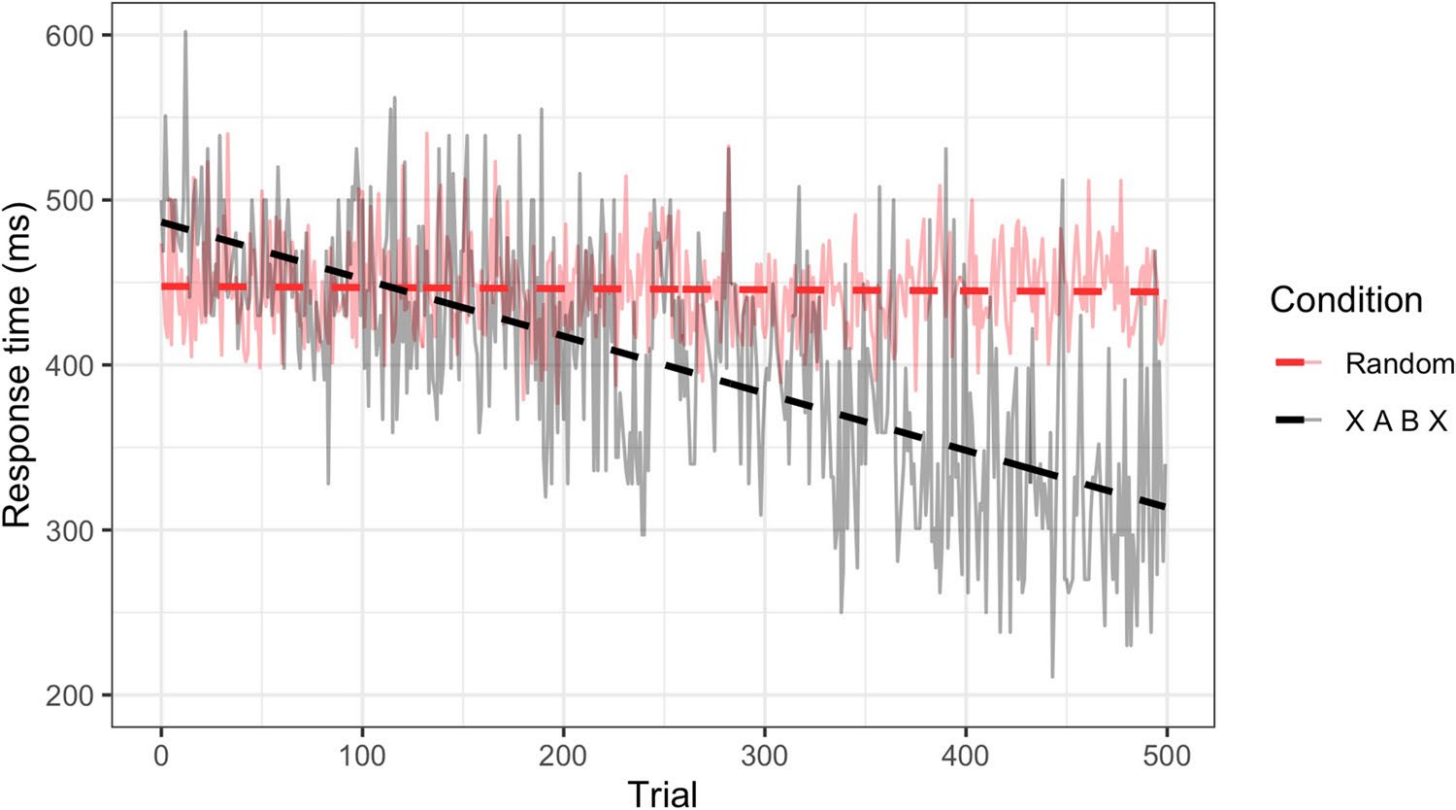
Example of experimental RTs with corresponding regression lines in the random (training) and XABX conditions (for the B element in an AB pair) for a single baboon

To measure the learning rate across repetitions of the AB regularity, we computed the slope of the regression line fitted to the RTs for the transition time from A to B (i.e., the RT on B) over the course of the 500 trials in each condition. Figure 2 provides an example of this procedure for one baboon and one experimental condition.

#### Analysis

We adopted a two-step trimming procedure. First, we excluded raw RTs greater than 800 ms. Second, RTs falling more than 2.5 standard deviations away from each baboon’s mean for a given block of 100 trials were subsequently excluded (9.88% of data excluded)^1^. With the remaining data, we performed two main analyses which produced convergent results. The first analyses were Bayesian repeated-measures ANOVA and the procedure is explained in the next section. The second analyses were linear mixed-effects regression analyses and they are reported in Appendix D.

##### Bayesian repeated-measures ANOVA

For each baboon, the slope was taken of the linear regression fit to the RTs for the transition from A to B over the 500 trials for each condition (ABXX, XABX, XXAB). For the baseline, we took the slope of the 500 random trials in the first block. This was done by taking the mean of all four touches in each trial, rendering one RT value per trial, and calculating the slope over all of these. The slopes were estimated using the *mldivide* function in the pracma package in R (Borchers, 2022). Once the slopes had been extracted, they were submitted to a Bayesian repeated-measures ANOVA with condition as the within-subjects factor, followed by post hoc pairwise Bayesian *t*-tests. We carried out another Bayesian repeated-measures ANOVA without the random baseline condition to examine whether there was any detectable difference between conditions. All Bayesian testing was carried out in the BayesFactor package for R (Morey & Rouder, 2021). We report Bayes factors (BF), which quantify the odds of the null hypothesis tested (difference of means = 0) compared with the alternative hypothesis (difference of means > 0). For example, a BF of 5 in favor of a given hypothesis means that given the evidence, that hypothesis is five times more likely than the alternative. BFs of 1 to 3 are considered weak evidence, BFs > 3 positive evidence, BFs > 20 strong evidence, and BFs > 150 very strong evidence (Raftery, 1995). Such Bayesian testing has the advantage of being able to present evidence for either the H_1_ (BF) or the H_0_ by taking the inverse of the Bayes factor (1/BF).

### Results

**Fig. 3:**
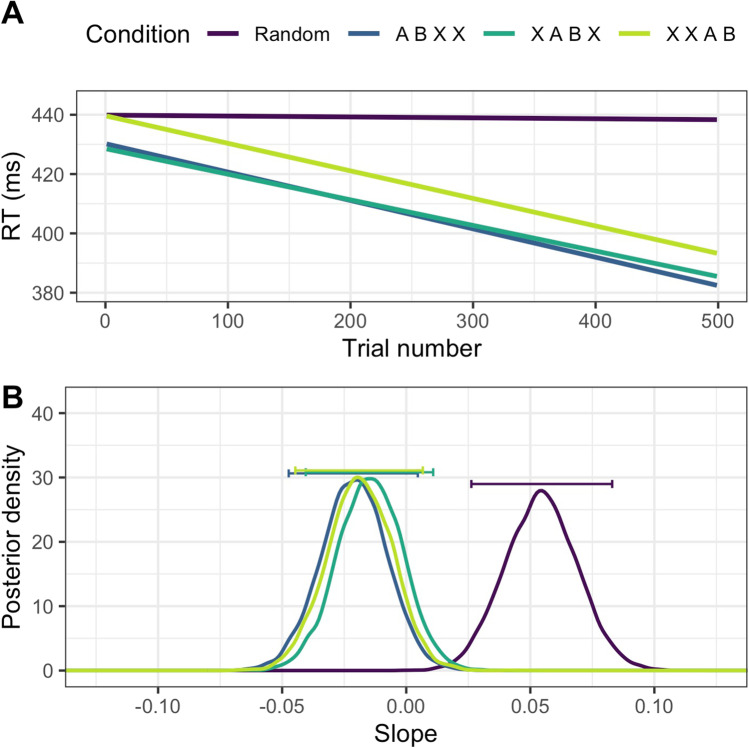
Results of Experiment 1. **A** Regression lines for the experimental conditions averaged over all participants. **B** Posterior distributions for slopes in the Random and three positional conditions. *Horizontal bars* show 95% of posterior estimates

Based on the results of the Bayesian comparison, there is decisive evidence that learning took place in all three of the regularity conditions relative to the random trials (BF = 97.18 ± 0.4%, Fig. 3). When the random condition was excluded, there was positive evidence for the null, i.e.: that there is no difference between the slopes in the regularity conditions (1/BF = 7.24 ± 0.77%)^2^. Each condition had a much steeper negative slope (arithmetic mean = – 0.091, – 0.086, and – 0.090 respectively; posterior maximum likelihood estimation (MLE), [95% credible interval (CI)] = – 0.021 [– .047, 0.005], – 0.015 [– 0.041, 0.011], and – 0.019 [– 0.045, 0.007] respectively) than the random condition (m = – 0.004; MLE = 0.05 [0.026, 0.083]). There is strong evidence that each of the conditions is different from the random baseline (indicating that learning took place; ABXX = 11.40 ± 0%, XABX = 15.05 ± 0%, XXAB = 126.07 ± 0%). We find, however, that the conditions do not differ from one another. We find that there is positive evidence for the null hypothesis between all of our learning conditions (1/BF; ABXX vs. XABX = 4.10 ± 0.02%, ABXX vs. XABX = 4.29 ± 0.02%, XABX vs. XXAB = 4.20 ± 0.02%).

### Discussion

In this first experiment, we found that the position of a simple AB regularity in a four-element sequence did not significantly impact the rate at which it is extracted. There was neither an advantage to having the regularity appear at the beginning or end of the trial, nor was there a crowding effect for the middle position, when A and B always appeared at the same positions.

These results suggest that the extraction of an AB regularity that is repeated on every trial at the same position in a sequence is not dependent upon its relative position in the sequence. The presence of random positions before or after the AB regularity does not impact its learning. The present data therefore provide a novel general property concerning associative learning mechanisms in sequences: the position of the regularity in the sequence does not matter.

If this general property is correct then changing the position of the regularity from trial to trial should not have an effect on the learning rate of the regularity. This was tested in Experiment 2 by using sequences of four elements including an AB regularity that appeared randomly at any possible position in the sequence on every trial. To test if the length of the sequence would impact the learning rate of the AB regularity, the same procedure was used with a sequence of five elements. Increasing the number of random elements within each trial may increase the interference produced by these random elements and slow down the extraction of the AB regularity.

## Experiment 2

### Method

#### Participants and apparatus

Twenty Guinea baboons (*Papio papio*, 13 females, age range, 4.42–25.25 years) completed this experiment. Sixteen of these also completed Experiment 1. The general task and apparatus used was the same as in Experiment 1.

#### Materials and procedure

The trial format was the same as in Experiment 1, with the exception that two sequence lengths (4 and 5) were presented to the baboons in two different blocks of 500 trials (the order of the blocks was counterbalanced across baboons). For each sequence length, baboons first saw 200 random trials for (re)familiarization with the task, followed by the 500 experimental trials. Whether the trial was four or five touches, the general format was the same. It contained a single two-element regularity (AB) and either two or three other random touches (X) drawn uniformly from the positions not used in the regularity, as it was the case in Experiment 1.

To avoid learning effects across conditions, each baboon had different regularities for the two sequence lengths and also different regularities from the ones they received in Experiment 1. These regularities were matched for difficulty based on the average RTs collected across the baboons during the random trials phase of Experiment 1. Two pairs were assigned to each baboon with the same constraints as in Experiment 1 and the list of pairs is presented in Appendix C.

In contrast to Experiment 1, instead of a given regularity appearing at the same position in each trial, the regularity appeared in a random position of the sequence on each trial. This was done first by evenly distributing the regularity over the three or four possible positions in the trial (i.e., ABXX, XABX, XXAB in the four-touch condition and ABXXX, XABXX, XXABX, XXXAB in the five-touch condition). This balanced list of trials was then shuffled such that the regularity could not appear in the same position for more than four trials in a row.

#### Analysis

We used the same exclusion criteria as in Experiment 1: Raw RTs greater than 800 ms were immediately excluded. RTs falling more than 2.5 standard deviations away from each baboon’s mean were subsequently excluded (18.55% of data excluded). Given the results of Experiment 1 (i.e., no significant differences between learning conditions), we aggregated across the three conditions from Experiment 1 and treated them as a single “Fixed position” condition in our analysis here.

##### Bayesian repeated-measures ANOVA

For each baboon, the slope was taken of the linear regression fit to the RTs for the transition from A to B over the trials in each condition (Fixed position from Experiment 1, Variable position – length 4, Variable position – length 5). For the baseline, we used the slope of the RTs from the 500 random trials in the first block of Experiment 1. The slopes were estimated using the mldivide function in the pracma package in R (Borchers, 2022). Once the slopes had been extracted, they were submitted to a Bayesian repeated-measures ANOVA with condition as the within-subjects factor, followed by post-hoc pairwise Bayesian *t*-tests. We carried out another Bayesian repeated-measures ANOVA without the random baseline condition to examine whether there was any detectable difference between conditions. All testing was carried out in the BayesFactor package for R (Morey & Rouder, 2021).

### Results

**Fig. 4:**
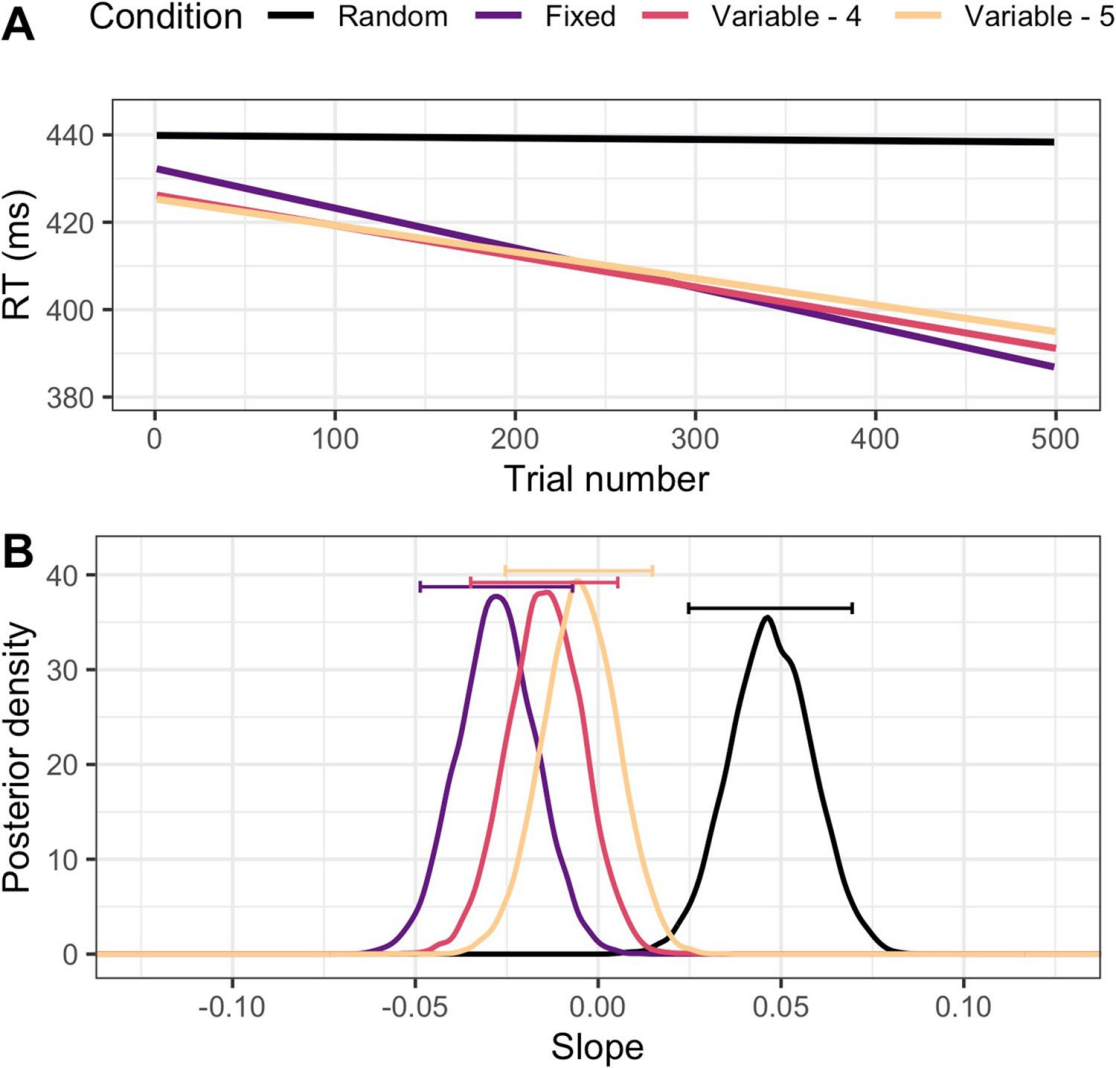
Results of Experiment 2. **A** Regression lines for the experimental conditions averaged over all participants. Note that the Fixed condition represents the aggregate of the three experimental conditions from Experiment 1. **B** Posterior distributions for slopes in the Random and experimental conditions. *Horizontal bars* show 95% of posterior estimates

As in Experiment 1, we found strong evidence that the means of all of our conditions were not equal in our omnibus test including the random baseline (BF = 733.61 ± 0.4%; Fig. 4). Each condition had a much steeper negative slope^3^ (arithmetic m_fix_ = – 0.090, m_var4_ = – 0.072, and m_var5_ = – 0.061; MLE_fix_ = – 0.028 [– 0.049, – 0.007], MLE_var4_ = – 0.015 [– 0.035, 0.005], MLE_var5_ = – 0.005 [– 0.025, 0.015]) than the random condition (m = – 0.004; MLE = 0.047 [0.025, 0.069]). When we conducted our test without the random baseline, we instead found positive evidence for H_0_: the conditions do not differ in learning rate (1/BF = 3.42 ± 0.61%). In the pairwise tests, we found strong evidence that each of the learning conditions (Fixed, Variable-4, and Variable-5) differed from the baseline (BF = 104.56 ± 0%, 111.27 ± 0%, and 53.94 ± 0%, respectively). In the pairwise tests between the learning conditions, we found evidence that the two length-four conditions do not differ (1/BF = 3.79 ± 0%), as well as that the two variable position conditions do not differ (1/BF = 3.94 ± 0.02%). The evidence for the relationship between the Fixed condition and the Variable-5 condition is weak, but in the direction of no difference between the conditions (1/BF = 2.04 ± 0%).

We note that the points included in the AB regularity occur at a much higher frequency (every trial) than the random points not included in the regularity (2/7 or 3/7 probability to occur in a given trial). To ensure that the learning effects observed in the RTs from A to B were not simply a function of the higher frequency of B relative to the randomly distributed points, we also examined the RT on A which had an equal frequency of appearance as B but had no predictable transition information from any other point on the grid in the variable position conditions. We thus conducted another Bayesian repeated-measures ANOVA which included the random baseline condition and the learning slope from the RT on the first element in the regularity (A) for the variable position conditions in our analysis.

We found positive evidence for the null hypothesis in this analysis (i.e., the learning rate on A was the same as in the random baseline condition; 1/BF = 7.10 ± 0.54%). In follow-up pairwise testing, we found that the learning rate on A in the variable position conditions did not differ either from the random baseline (1/BF = 3.42 ± 0% and 3.17 ± 0% for Var-4 and Var-5 against baseline, respectively), or from each other (1/BF = 4.26 ± 0.02%). We also found strong evidence that the learning rate on A is different from that on B in both of these conditions (BF = 91.77 ± 0% and 1685.68 ± 0%, respectively). We can interpret this as confirmation that the learning observed on B in the AB regularity is truly a function of its relationship to A, and not simply a question of relative frequency.

### Discussion

In this second experiment, we first found that when the position of the regularity was variable from trial to trial, it was reliably extracted and, more importantly, the learning rate in this variable condition was similar to the fixed condition from Experiment 1. Second, increasing the length of the sequence (and therefore the number of random elements) did not have an effect on the learning rate of the AB regularity.

These results confirm the general property obtained in Experiment 1: the position of the AB regularity in the sequence does not matter. Its repeated occurrence on every trial determines its learning independently of its position in the sequence. The manipulation of sequence length also suggests another general property of associative learning mechanisms: length does not seem to affect regularity extraction. However, this second claim is restricted to the sequence length we manipulated here. It remains possible that with a longer sequence, learning rate could be adversely impacted.

We also confirmed that the learning observed on B was not merely an effect of increased frequency relative to other positions, as it had exactly the same appearance frequency as A, but A was not learned, while B was. We thus provide new empirical evidence that there is no effect of these manipulations on associative learning in this context.

## General discussion

The present study was designed to investigate three simple questions about the extraction of simple AB regularities. The first question was about the role of the position of the regularity in the sequence and more specifically, if there was a difference in the extraction of the regularity when it occurred systematically at the beginning, middle, or final position in a sequence composed of four elements. The second question concerned the fixed or variable position of the AB regularity in the sequence and whether it influences regularity extraction. The third question was about the role of sequence length in the learning of the AB regularity.

First, in Experiment 1, AB was inserted at a fixed position in a four-element sequence, either before, after or between two random elements. The AB dependency was progressively learned by baboons, but no differences were observed on the learning rates in these three conditions. This first result indicates that the absolute position of the dependency relative to the noise does not affect the extraction of the dependency itself, suggesting the general property that, during serial learning, individuals learn the relationship between adjacent elements AB independently of the position of the regularity within the sequence.

A second main result stems from Experiment 2 and the manipulation of the position of AB within the sequence. In this second experiment, the position of AB (before, after or between random elements) varied across trials and its position relative to the noise was not a reliable information anymore. Under these conditions, the AB dependency was still extracted and learned by baboons, suggesting that coding for position was not necessary and that varying positions did not hinder learning. Furthermore, the learning rates between fixed and varying positions of AB were not different, suggesting that, even in a fixed design, coding of ordinal positions either does not facilitate learning of a simple AB dependency, or did not even occur at all.

Our third main result deals with the position and amount of noise in the sequence. As already stated, in a 1:1 ratio of signal to noise (i.e., when there are two random elements and two regular elements AB), the position of the regularity relative to the noise did not impact the learning of AB. However, this absence of effect remained even with a decreased signal-to-noise ratio (i.e., three random elements), indicating how salient a simple AB dependency was in a random environment.

Taken together, these results provide relatively simple answers to the three questions addressed in these two experiments and also provide novel empirical constraints for computational models of statistical learning. First, these data suggest that models probably don’t need to code for the ordinal position of an AB regularity in a sequence and that learning occurs with the same strength when the regularity is positioned at the beginning, middle, or final position in a sequence. Second, the absence of a difference in the extraction of a regularity appearing in a fixed or variable position suggests a predominance of adjacency coding mechanisms over serial position coding ones during serial learning. Third, since the number of random elements in the sequence, and consequently the signal-to-noise ratio and length of the sequence, did not hinder learning, this suggests that the critical feature for extracting simple AB regularities is mainly their repetition.

These results are generally consistent with chunking models of statistical learning (e.g., Perruchet & Vinter, 1998) for which regularity extraction is insensitive to the present set of manipulated variables (i.e., position, fixed or variable, sequence length).

The generality of these conclusions needs, however, to be confirmed by additional empirical evidence. Indeed, the variation in sequence length is relatively limited in the present study since we only compared learning for sequences of four and five elements. Increasing sequence length in Experiment 1 may produce some difference between the three fixed positions tested in this experiment. With longer sequences, the extraction of the AB regularity positioned in the middle of the sequence might be harder. Similarly, increasing sequence length in Experiment 2 may slow down the extraction of the regularity. However, if the same learning dynamics of AB regularities is observed for longer sequences, then clearly, it will indicate that the main ingredients for computational models of statistical learning are 1) repetition and 2) adjacency coding mechanisms.

In sum, the present set of results suggests that regularity extraction for simple AB repeated associations is not influenced 1) by the position of the regularity within the sequence; 2) by its variable or fixed occurrence in the sequence; and 3) by the length of the sequence. Unless new data show otherwise, especially with longer sequences, these three results provide clear and general empirical constraints for current models of statistical learning.

### Supplementary information

Below is the link to the electronic supplementary material.

Supplementary file1 (PDF 367 KB)

## Footnotes

1 Inspection of the response times distribution revealed that a majority of responses were produced around 500 ms. A smaller group of RTs appeared around 1000 ms and was likely due to situations in which baboon’s response was not recorded by the computer, because their hands were dirty. In this situation, they had to touch the screen again, and longer RTs were recorded (that are on average twice longer compared to the first responses). This is why we have adopted this two-step trimming procedure.

2 By taking the inverse of the Bayes factor (1/BF), we can examine the evidence for the null hypothesis (μ_1_= μ_2_).

3 Observed slopes for both experiments are plotted in Supplementary Figure 1.
