# Supplementary material for: Simple questions on simple associations: regularity extraction in non-human primates
Source: Learn Behav. 2023 Jun 7;51(4):392–401. doi: 10.3758/s13420-023-00579-z (PMC10716064; doi:10.3758/s13420-023-00579-z)
Supplement: Supplementary file 1 — Supplementary file1 (PDF 367 KB) [file 13420_2023_579_MOESM1_ESM.pdf]

Appendixes

Supplementary Figures

Supplementary Figure 1: Observed slopes by condition for Experiments 1 & 2

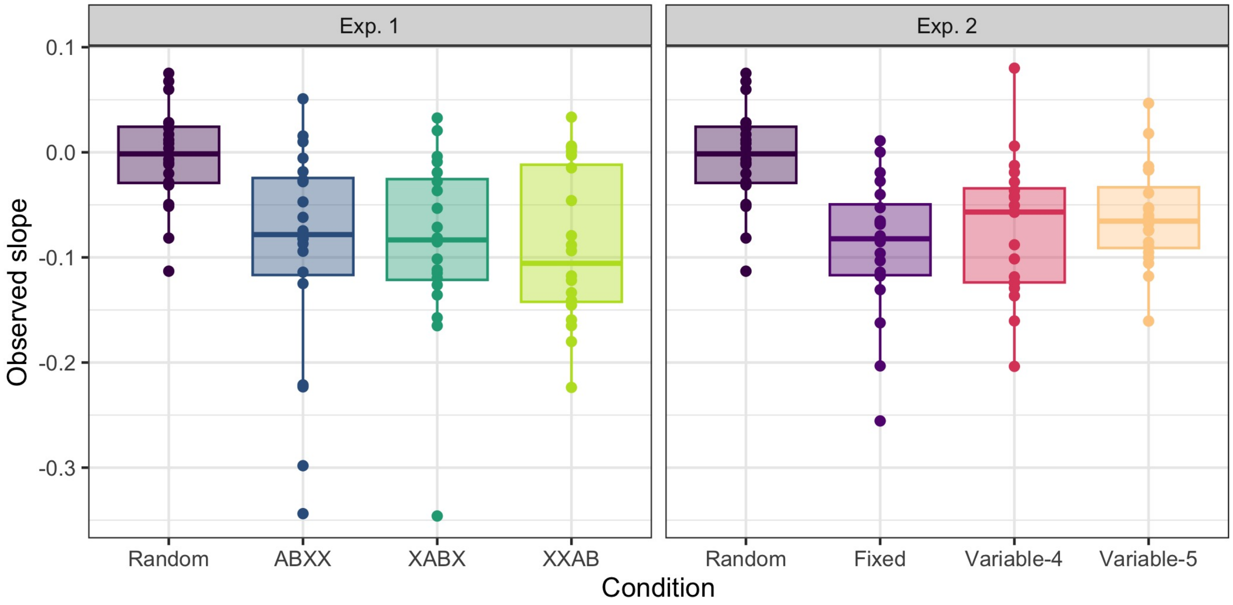

Figure 1: Box plots of observed slopes in Experiments 1 and 2. Points represent values for individual baboons.

Appendix A

Mean response times over the entire group of baboons for each of the 72 possible transitions calculated from the 1000 random trials

start	1	2	3	4	5	6	7	8	9
1	NA	519	573	495	482	509	521	497	543
2	569	NA	553	513	474	511	523	491	509
3	558	519	NA	513	472	488	544	493	512
4	551	517	560	NA	464	509	522	482	546
5	549	504	552	501	NA	483	535	479	527
6	567	515	546	507	484	NA	533	483	511
7	555	504	558	475	463	516	NA	484	541
8	554	512	540	485	448	472	512	NA	507
9	546	512	540	514	460	464	550	485	NA

Note. All transitions are in milliseconds (ms) and correspond to the time elapsed between the disappearance of the red circle from the 1st position of the Transition (rows) and the monkey's touch on the 2nd position of the Transition (columns).

Appendix B

Repartition of AB pairs learned by each baboon in Experiment 1 and corresponding baseline mean transition times

Name	ID	Position order	ABXX	ABXX baseline	XABX	XABX baseline	XXAB	XXAB baseline
ANGELE	1	2, 3, 1	8, 7	512	9, 2	512	4, 6	509
ARIELLE	2	3, 2, 1	1, 7	521	3, 9	512	6, 2	515
ATMOSPHERE	3	2, 3, 1	3, 6	488	1, 5	482	4, 8	482
DORA	4	3, 1, 2	6, 4	507	2, 9	509	8, 7	512
DREAM	5	1, 2, 3	9, 2	512	1, 6	509	3, 4	513
EWINE	6	2, 1, 3	6, 8	483	7, 4	475	2, 5	474
FANA	7	3, 1, 2	6, 3	546	4, 9	546	5, 1	549
FELIPE	8	3, 1, 2	8, 2	512	3, 9	512	4, 6	509
FEYA	9	1, 3, 2	4, 1	551	2, 3	553	9, 7	550
FLUTE	10	2, 3, 1	6, 9	511	3, 2	519	8, 7	512
HARLEM	11	2, 1, 3	3, 7	544	4, 9	546	8, 1	554
KALI	12	3, 2, 1	1, 7	521	3, 9	512	6, 2	515
LIPS	13	1, 2, 3	3, 2	519	8, 7	512	4, 6	509
LOME	14	2, 1, 3	5, 2	504	8, 9	507	3, 4	513
MAKO	15	1, 3, 2	6, 4	507	5, 2	504	1, 8	497
MALI	16	1, 3, 2	3, 4	513	6, 9	511	1, 7	521
MUSE	17	2, 1, 3	5, 4	501	1, 6	509	8, 9	507
NEKKE	18	1, 2, 3	6, 9	511	4, 2	517	1, 7	521
PETOULETTE	19	2, 3, 1	6, 9	511	8, 2	512	1, 7	521
VIOLETTE	20	3, 1, 2	9, 4	514	1, 6	509	3, 2	519

Note. **Position order** column indicates the order in which the baboon was presented with the conditions where 1 = ABXX, 2 = XABX, and 3 = XXAB. For example, if the position order was 2, 3, 1, then the baboon saw the conditions as XABX followed by XXAB followed by ABXX.

Appendix C

Repartition of AB pairs learned by each baboon in Experiment 2 and corresponding baseline mean transition times

Name	ID	Var-4	Var-4 baseline	Var-5	Var-5 baseline
ANGELE	1	6, 4	448	1, 7	447
ARIELLE	2	6, 9	424	3, 5	418
ATMOSPHERE	3	1, 2	463	4, 7	457
EWINE	6	9, 7	438	3, 6	438
FANA	7	1, 5	414	2, 8	404
FELIPE	8	4, 6	427	9, 2	438
FEYA	9	6, 4	448	8, 7	451
HARLEM	11	8, 4	433	5, 9	433
KALI	12	6, 7	447	8, 3	445
LIPS	13	4, 8	420	5, 6	424
LOME	14	1, 7	447	5, 4	446
MAKO	15	8, 9	446	4, 2	449
MALI	16	3, 4	449	8, 7	451
MUSE	17	9, 1	461	6, 2	462
PETOULETTE	19	2, 6	441	7, 3	441
VIOLETTE	20	7, 5	405	2, 8	404
ARTICHO	21	5, 6	424	4, 8	420
BOBO	22	8, 9	446	3, 7	443
HERMINE	23	9, 4	430	1, 6	429
PIPO	24	5, 3	460	1, 2	463

Appendix D

Results from Linear Mixed-Effects modeling analysis

The same model was fit with each of the four conditions (`cond`) from Experiment 1 as the baseline:

Variable name	Condition name
rnd	random baseline condition
pos1	ABXX
pos2	XABX
pos3	XXAB

Because models which used `trial number` as a variable did not converge, trials were grouped into groups of 20 and this is the slope term in the models (`trl20`).

For the other model terms, `rtVal` is the response time in milliseconds, `name` is the identifier for each baboon, and `ptPair` are the unique pairs of start and stop points on the screen.

In each case, we find that a) all conditions have a negative slope above chance except the random condition, and b) the regularity conditions (ABXX, XABX, XXAB) differ from the random baseline condition, but do not significantly differ from one another. While this presents promising evidence, the absence of an effect is not the same as evidence for the null, which is why we switched our analysis over to the Bayesian framework.

Random condition as baseline

```
## Linear mixed model fit by REML. t-tests use Satterthwaite's method [
## lmerModLmerTest]
## Formula: rtVal ~ cond * trl20 + (trl20 + cond | name) * (1 | ptPair)
##    Data: .
## Control: lmerControl(optimizer = "bobyqa", optCtrl = list(maxfun = 1e+05))
##
## REML criterion at convergence: 647288.7
##
## Scaled residuals:
##     Min      1Q  Median      3Q     Max
## -3.8971 -0.6658 -0.1238  0.5115  6.0512
##
## Random effects:
##  Groups   Name        Variance  Std.Dev. Corr
##  ptPair   (Intercept)  459.9602 21.4467
##  name     (Intercept)  356.7099 18.8868
##           trl20          0.4932  0.7023  -0.33
##           condpos1     729.7698 27.0143   0.15  0.33
##           condpos2     509.6477 22.5754  -0.08 -0.06  0.49
##           condpos3     923.9344 30.3963  -0.19 -0.06 -0.16 -0.28
##  Residual             2380.6093 48.7915
## Number of obs: 60916, groups:  ptPair, 81; name, 20
##
## Fixed effects:
##                  Estimate Std. Error         df t value Pr(>|t|)
## (Intercept)     4.407e+02  4.878e+00  3.235e+01  90.349   <2e-16 ***
## condpos1       -1.530e+01  6.178e+00  2.010e+01  -2.477   0.0222 *
## condpos2       -1.076e+01  5.218e+00  2.075e+01  -2.062   0.0519 .
## condpos3       -6.420e+00  6.934e+00  1.993e+01  -0.926   0.3656
## trl20          -6.632e-02  1.614e-01  1.993e+01  -0.411   0.6854
## condpos1:trl20 -1.792e+00  7.984e-02  6.074e+04 -22.450   <2e-16 ***
## condpos2:trl20 -1.657e+00  8.062e-02  6.074e+04 -20.553   <2e-16 ***
## condpos3:trl20 -1.751e+00  8.212e-02  6.074e+04 -21.323   <2e-16 ***
## ---
## Signif. codes:  0 '***' 0.001 '**' 0.01 '*' 0.05 '.' 0.1 ' ' 1
##
## Correlation of Fixed Effects:
##             (Intr) cndps1 cndps2 cndps3 trl20  cn1:20 cn2:20
## condpos1     0.120
## condpos2    -0.081  0.471
## condpos3    -0.169 -0.142 -0.254
## trl20       -0.298  0.329 -0.035 -0.045
## cndps1:tr20  0.043 -0.156 -0.040 -0.030 -0.106
## cndps2:tr20  0.042 -0.034 -0.186 -0.030 -0.105  0.213
## cndps3:tr20  0.041 -0.033 -0.039 -0.143 -0.103  0.208  0.206
```

ABXX condition as baseline

```
## Linear mixed model fit by REML. t-tests use Satterthwaite's method [
## lmerModLmerTest]
## Formula: rtVal ~ cond * trl20 + (trl20 + cond | name) + (1 | ptPair)
##    Data: .
## Control: lmerControl(optimizer = "bobyqa", optCtrl = list(maxfun = 1e+05))
##
## REML criterion at convergence: 647288.7
##
## Scaled residuals:
##     Min      1Q  Median      3Q     Max
## -3.8971 -0.6658 -0.1238  0.5115  6.0512
##
## Random effects:
##  Groups   Name        Variance  Std.Dev. Corr
##  ptPair   (Intercept)  459.9562 21.4466
##  name     (Intercept) 1241.8690 35.2402
##           trl20          0.4932  0.7023   0.08
##           condpos2     645.7056 25.4107  -0.61 -0.40
##           condpos3    1911.8915 43.7252  -0.68 -0.25  0.34
##           condrnd      729.5335 27.0099  -0.85 -0.33  0.63  0.73
##  Residual             2380.6095 48.7915
## Number of obs: 60916, groups:  ptPair, 81; name, 20
##
## Fixed effects:
##                  Estimate Std. Error         df t value Pr(>|t|)
## (Intercept)     4.254e+02  8.316e+00  2.306e+01  51.156  < 2e-16 ***
## condpos2        4.541e+00  5.920e+00  2.070e+01   0.767   0.4517
## condpos3        8.883e+00  9.919e+00  1.967e+01   0.896   0.3813
## condrnd         1.530e+01  6.177e+00  2.012e+01   2.478   0.0222 *
## trl20          -1.859e+00  1.722e-01  2.588e+01 -10.792 4.49e-11 ***
## condpos2:trl20  1.354e-01  1.007e-01  6.074e+04   1.345   0.1786
## condpos3:trl20  4.127e-02  1.019e-01  6.074e+04   0.405   0.6855
## condrnd:trl20   1.792e+00  7.984e-02  6.074e+04  22.450  < 2e-16 ***
## ---
## Signif. codes:  0 '***' 0.001 '**' 0.01 '*' 0.05 '.' 0.1 ' ' 1
##
## Correlation of Fixed Effects:
##             (Intr) cndps2 cndps3 cndrnd trl20  cn2:20 cn3:20
## condpos2    -0.582
## condpos3    -0.649  0.338
## condrnd     -0.813  0.629  0.722
## trl20        0.023 -0.290 -0.186 -0.235
## cndps2:tr20  0.072 -0.204 -0.060 -0.097 -0.289
## cndps3:tr20  0.071 -0.100 -0.124 -0.096 -0.285  0.488
## cndrnd:tr20  0.091 -0.128 -0.076 -0.156 -0.364  0.623  0.616
```

XABX condition as baseline

```
## Linear mixed model fit by REML. t-tests use Satterthwaite's method [
## lmerModLmerTest]
## Formula: rtVal ~ cond * trl20 + (trl20 + cond | name) + (1 | ptPair)
##    Data: .
## Control: lmerControl(optimizer = "bobyqa", optCtrl = list(maxfun = 1e+05))
##
## REML criterion at convergence: 647288.7
##
## Scaled residuals:
##     Min      1Q  Median      3Q     Max
## -3.8971 -0.6658 -0.1238  0.5115  6.0512
##
## Random effects:
##  Groups   Name        Variance  Std.Dev. Corr
##  ptPair   (Intercept)  459.9527 21.4465
##  name     (Intercept)  795.7343 28.2088
##           trl20          0.4932  0.7023  -0.27
##           condpos3    1813.0592 42.5800  -0.64 -0.01
##           condrnd      509.7522 22.5777  -0.74  0.06  0.73
##           condpos1     645.8306 25.4132  -0.14  0.40  0.25  0.37
##  Residual             2380.6090 48.7915
## Number of obs: 60916, groups:  ptPair, 81; name, 20
##
## Fixed effects:
##                  Estimate Std. Error         df t value Pr(>|t|)
## (Intercept)     4.300e+02  6.851e+00  2.547e+01  62.762  < 2e-16 ***
## condpos3        4.342e+00  9.682e+00  1.974e+01   0.448   0.6587
## condrnd         1.076e+01  5.219e+00  2.074e+01   2.062   0.0519 .
## condpos1       -4.541e+00  5.921e+00  2.069e+01  -0.767   0.4517
## trl20          -1.723e+00  1.726e-01  2.610e+01  -9.983 2.11e-10 ***
## condpos3:trl20 -9.416e-02  1.025e-01  6.074e+04  -0.918   0.3584
## condrnd:trl20   1.657e+00  8.062e-02  6.074e+04  20.553  < 2e-16 ***
## condpos1:trl20 -1.354e-01  1.007e-01  6.074e+04  -1.345   0.1786
## ---
## Signif. codes:  0 '***' 0.001 '**' 0.01 '*' 0.05 '.' 0.1 ' ' 1
##
## Correlation of Fixed Effects:
##             (Intr) cndps3 cndrnd cndps1 trl20  cn3:20 cnd:20
## condpos3    -0.605
## condrnd     -0.704  0.721
## condpos1    -0.158  0.265  0.390
## trl20       -0.275  0.024  0.119  0.409
## cndps3:tr20  0.088 -0.127 -0.115 -0.101 -0.290
## cndrnd:tr20  0.111 -0.079 -0.186 -0.129 -0.369  0.621
## cndps1:tr20  0.089 -0.063 -0.117 -0.204 -0.295  0.497  0.632
```

XXAB condition as baseline

```
## Linear mixed model fit by REML. t-tests use Satterthwaite's method [
## lmerModLmerTest]
## Formula: rtVal ~ cond * trl20 + (trl20 + cond | name) + (1 | ptPair)
##    Data: .
## Control: lmerControl(optimizer = "bobyqa", optCtrl = list(maxfun = 1e+05))
##
## REML criterion at convergence: 647288.7
##
## Scaled residuals:
##     Min      1Q  Median      3Q     Max
## -3.8971 -0.6658 -0.1238  0.5115  6.0512
##
## Random effects:
##  Groups   Name        Variance  Std.Dev. Corr
##  ptPair   (Intercept)  459.9519 21.4465
##  name     (Intercept) 1062.9213 32.6025
##           trl20          0.4933  0.7023  -0.25
##           condrnd      923.8909 30.3956  -0.82  0.06
##           condpos1    1912.3018 43.7299  -0.61  0.25  0.79
##           condpos2    1812.5847 42.5745  -0.75  0.01  0.86  0.83
##  Residual             2380.6092 48.7915
## Number of obs: 60916, groups:  ptPair, 81; name, 20
##
## Fixed effects:
##                 Estimate Std. Error        df t value Pr(>|t|)
## (Intercept)     4.343e+02  7.773e+00  2.376e+01  55.872  < 2e-16 ***
## condrnd         6.420e+00  6.934e+00  1.994e+01   0.926    0.366
## condpos1       -8.883e+00  9.920e+00  1.966e+01  -0.896    0.381
## condpos2       -4.342e+00  9.681e+00  1.975e+01  -0.448    0.659
## trl20          -1.817e+00  1.733e-01  2.654e+01 -10.485 6.21e-11 ***
## condrnd:trl20   1.751e+00  8.212e-02  6.074e+04  21.323  < 2e-16 ***
## condpos1:trl20 -4.127e-02  1.019e-01  6.074e+04  -0.405    0.686
## condpos2:trl20  9.416e-02  1.025e-01  6.074e+04   0.918    0.358
## ---
## Signif. codes:  0 '***' 0.001 '**' 0.01 '*' 0.05 '.' 0.1 ' ' 1
##
## Correlation of Fixed Effects:
##             (Intr) cndrnd cndps1 cndps2 trl20  cnd:20 cn1:20
## condrnd     -0.786
## condpos1    -0.581  0.787
## condpos2    -0.712  0.853  0.818
## trl20       -0.260  0.110  0.257  0.051
## cndrnd:tr20  0.101 -0.143 -0.079 -0.082 -0.378
## cndps1:tr20  0.082 -0.092 -0.124 -0.066 -0.305  0.643
## cndps2:tr20  0.081 -0.091 -0.064 -0.127 -0.303  0.639  0.515
```
